# Inevitable isolation and the change of stress markers in hemodialysis patients during the 2015 MERS-CoV outbreak in Korea

**DOI:** 10.1038/s41598-019-41964-x

**Published:** 2019-04-05

**Authors:** Yang Gyun Kim, Haena Moon, Se-Yun Kim, Yu-Ho Lee, Da-Wun Jeong, Kipyo Kim, Ju Young Moon, Young-Ki Lee, Ajin Cho, Hong-Seock Lee, Hayne Cho Park, Sang-Ho Lee

**Affiliations:** 1 Division of Nephrology, Kyung Hee University College of Medicine, Seoul, Korea; 2 Hallym University College of Medicine, Internal Medicine, Seoul, Korea; 3 Hallym University College of Medicine, Psychiatry, Seoul, Korea; 4 Department of Internal Medicine, Armed Forces Capital Hospital, Seongnam, Korea

## Abstract

During the outbreak of Middle East respiratory syndrome coronavirus(MERS-CoV) in 2015, one hemodialysis patient was infected with MERS-CoV, and the remaining hemodialysis(HD) patients (n = 83) and medical staff (n = 12) had to undergo dialysis treatment in an isolated environment. This study was performed to investigate the effects of stress caused by dialysis treatment under isolation. Plasma samples from the HD patients and medical staff were collected at the time of isolation(M0), the following month(M1), and three months after isolation(M3). Parameters for stress included circulating cell-free genomic DNA(ccf-gDNA), circulating cell-free mitochondria DNA(ccf-mtDNA), and pentraxin-3(PTX-3). Decreased values of Hct, kt/v and ca x p were recovered after the end of two weeks of isolation. The levels of ccf-gDNA and ccf-mtDNA were the highest at M0 and decreased gradually in both HD patients and the medical staff. The normalization of ccf-gDNA and ccf-mtDNA was significantly delayed in HD patients compared with the response in the medical staff. PTX-3 increased only in HD patients and was highest at M0, and it then gradually decreased. Medical isolation and subnormal quality of care during the MERS outbreak caused extreme stress in HD patients. Plasma cell-free DNA and PTX-3 seems to be good indicators of stress and quality of care in HD patients.

## Introduction

The HD unit was the principal location for the propagation of MERS infections in 2013 in Saudi Arabia^1^. One patient on maintenance hemodialysis (HD) in our hospital was infected with Middle East respiratory syndrome coronavirus (MERS-CoV) during the outbreak of MERS in Korea. The 83 HD patients and 12 medical staff were exposed to the MERS patient. Facing the possibility of a large epidemic, we had to follow the guidelines by National Disaster Management; thus, we decided to quarantine all exposed HD patients and medical staff in the hospital or their home. The medical staff caring for those patients had to wear level D personal protection equipment during work and were isolated in their homes after work^2^. Patients who were quarantined irrespective of their wishes suffered from isolation and fear of infection. Fortunately, no additional patients were infected with MERS-CoV, but all HD patients and medical staff experienced extreme physical and mental stress for the 17 days of isolation.

Circulating cell-free DNA (circulating-cfDNA) is released from damaged cells and associated with various pathological conditions^3,4^. Previous studies showed physical or emotional stress could augment circulating-cfDNA^5,6^. Also, circulating-cfDNA was a predictor of mortality and showed a close association with chronic inflammation in HD patients^7,8^. Pentraxin-3 (PTX-3) is a marker of systemic inflammation producing rapidly by various cells under the inflammation or stress conditions^9^. These three parameters are commonly used as stress parameters in pathologic status.

We thought sudden isolation could lead to severe stress and influence the clinical outcome of HD patients. So, the clinical data and serum sample was collected in the beginning and middle of the isolation period and a long time after isolation. This study was designed to confirm our hypothesis that MERS isolation could affect not only clinico-laboratory findings but also various stress markers including circulating cf-DNA and PTX-3.

## Results

### Clinico-laboratory findings in HD patients

**Figure 1:**
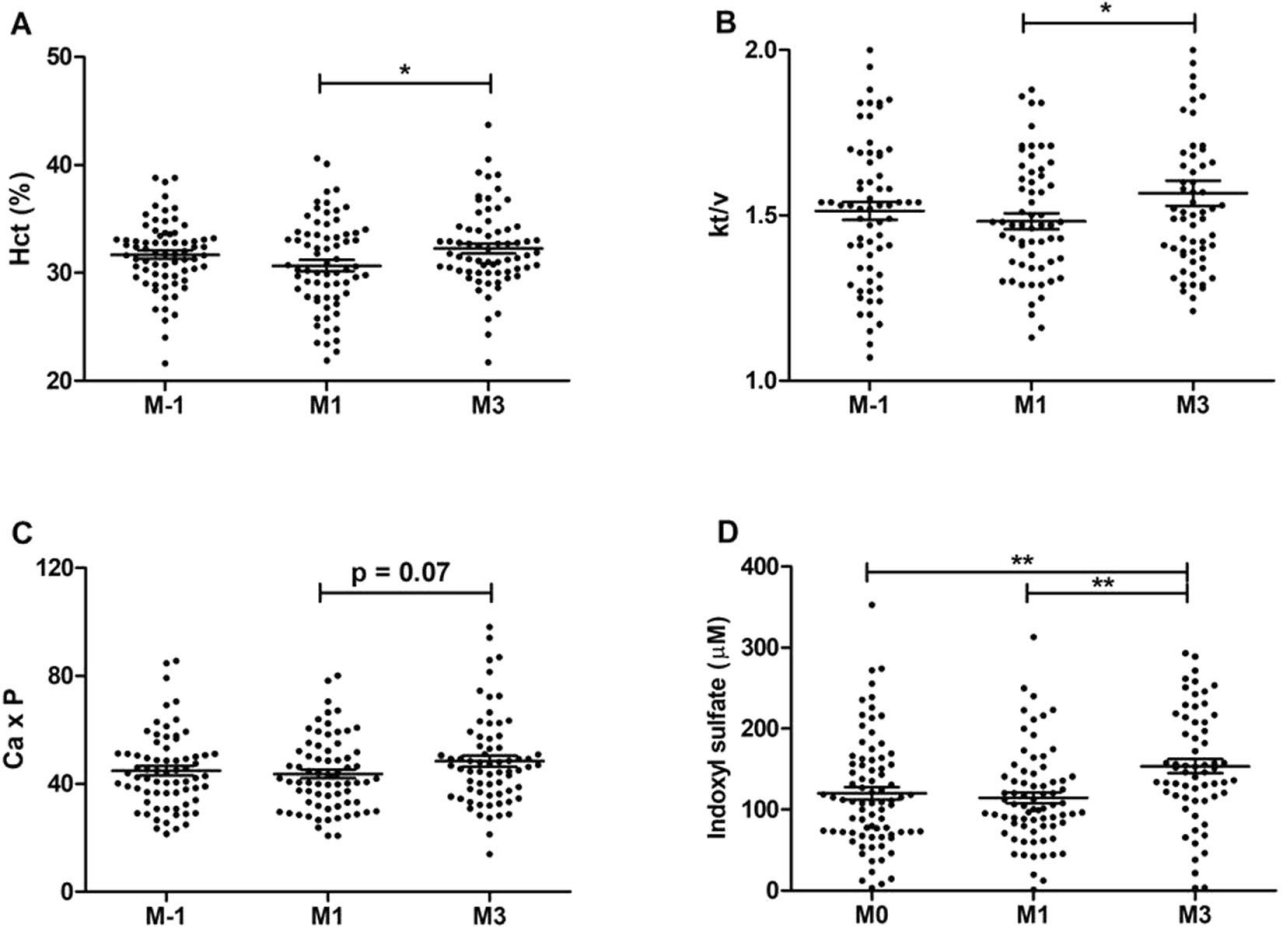
Changes in laboratory findings and indoxyl sulfate before and after MERS isolation (**A**) Hct, (**B**) kt/v, and (**C**) ca x p were decreased at M1 and recovered after MERS isolation. (**D**) The level of indoxyl sulfate was similar at M0 and M1 but significantly increased at M3. For Hct and kt/v, *P value < 0.05 vs. M1 (ANOVA, LDS post-hoc analysis) For indoxyl sulfate, **P value < 0.01.

Forty-five percent of the patients were undergoing dialysis because of diabetes mellitus end-stage renal disease (DM-ESRD) (Table 1). The average value of the Charlson comorbidity index was 4.52 ± 1.86. The Hct were decreased at M1, then recovered at M3 (Fig. 1, Table 2). The level of kt/v at M1 was lower than the levels before and after the MERS isolation period, but a significant difference was found only between the level of M1 and M3. The indoxyl sulfate (IS) level was not decreased at M0-M1. The levels of IS at M3 were significantly higher than those at M0 and M1 (Fig. 1). The levels of IS at M0 closely correlated with those at M1 and M3 (Supplementary Fig. S1). Two HD patients died during quarantine. The first death was ascribed to aspiration pneumonia in an 88-year-old female patient with hypertensive ESRD. The second death was caused by mechanical ileus and pneumonia in a 91-year-old female with hypertensive ESRD.

**Table 1:** Baseline characteristics in HD patients and medical staff.

	HD patients (n = 83)	Medical staffs (n = 12)	p-value
Age	62.09 ± 1.54	36.01 ± 1.61	<0.001
Sex (M/F)	48/35	0/12	<0.001
Charlson comorbidity intex	4.52 ± 1.86	(nurses 10, doctors 2)	
BMI (kg/m^2^)	24.16 ± 0.38		
Dry weight (kg)	62.79 ± 1.06		
SBP/DBP (mmHg)	141.41 ± 2.06/74.24 ± 1.44		
Dialysis vintage (month)	74.24 ± 1.44		
Cause of ESRD			
DM	38/83 (45.78%)		
Hypertension	15/83 (18.07%)		
Glomerulonephritis	14/83 (16.87%)		
Others	12/83 (14.46%)		
Unknown	4/83 (4.82%)		

BMI, body mass index; SBP, systolic blood pressure; DBP, diastolic blood pressure; ESRD, end stage renal disease; DM, diabetes mellitus.

**Table 2:** Changes in laboratory findings before and after MERS isolation.

	M-1	M1	M3	p-value
Hb (g/dL)	10.28 ± 1.07	10.15 ± 1.40	10.51 ± 1.67	0.305
**Hct (%)**	**31.68 **±** 3.22**	**30.67 **±** 4.44**	**32.26 **±** 3.74**	**0.049**
**Ca (mg/dL)**	**8.08 **±** 0.86**	**8.27 **±** 0.75**	**8.53 **±** 0.92**	**0.008**
P (mg/dL)	5.54 ± 1.52	5.29 ± 1.62	5.65 ± 1.71	0.418
CaxP	44.84 ± 14.06	43.64 ± 13.52	48.40 ± 16.76	0.150
intact PTH (pg/mL)	362.96 ± 186.15	364.01 ± 209.63	392.93 ± 231.71	0.697
Albumin (g/dL)	3.58 ± 0.39	3.63 ± 0.34	3.67 ± 0.28	0.315
hs CRP (mg/dL)	5.11 ± 11.15	4.78 ± 8.44	6.75 ± 15.82	0.675
Total cholesterol (mg/dL)	142.29 ± 4.43	137.80 ± 4.51	150.26 ± 5.51	0.313
Iron (µg/dL)	63.46 ± 26.89	72.71 ± 41.19	75.03 ± 35.71	0.148
TIBC (µg/dL)	209.89 ± 37.82	221.94 ± 95.29	227.41 ± 35.42	0.283
Ferritin (ng/dL)	451.62 ± 281.88	443.98 ± 324.83	371.45 ± 339.72	0.281
Kt/v	1.51 ± 0.22	1.48 ± 0.19	1.57 ± 0.29	0.137

Hb, hemoglobin; Hct, hematocrit; Ca, calcium; P, phosphorus; intact PTH, intact parathyroid hormone; TIBC, total iron-binding capacity.

We used single pool Kt/V to indicate dialysis adequacy.

### Effect of isolation on the level of ccf-gDNA

**Figure 2:**
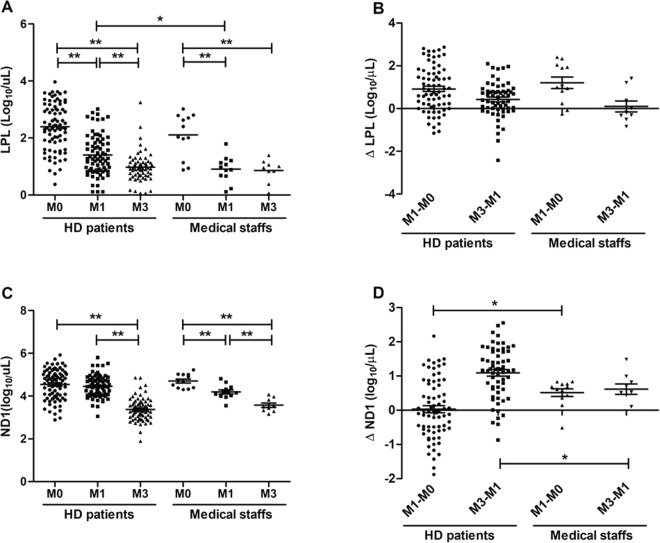
Log levels of ccf-gDNA and ccf-mtDNA at M0, M1, and M3 The human lipoprotein lipase gene (LPL) for gDNA and the human NADH1 dehydrogenase 1 gene (ND1) for mtDNA were used. (**A**) The level of ccf-gDNA was highest at M0 in both HD patients and medical staff and then gradually decreased. However, the recovery was delayed in HD patients. (**B**) The delta values of ccf-gDNA between M0 and M1 and between M3 and M1 were similar. (**C**) The level of ccf-mtDNA was also highest at M0 in patients and medical staff and decreased for 3 months. (**D**) ccf-mtDNA was decreased between M0 and M1 but decreased less between M3 and M1 in medical staff than in HD patients. *P value < 0.05 and ** < 0.01.

Segregation affected the levels of circulating cf-DNA. The ccf-gDNA (LPL) was significantly elevated at M0, irrespective of whether the subject was a patient or staff member (Fig. 2). The levels of ccf-gDNA decreased over time, but the decrease was delayed in HD patients. At M1, ccf-gDNA levels were significantly higher in HD patients than in the medical staff. The ccf-gDNA level initially abruptly decreased in medical staff but slowly reduced in HD patients. The delta value of ccf-gDNA between M0 and M1 and between M1 and M3 was not different between HD patients and medical staff. These results indicated that stress was sufficient to increase ccf-gDNA levels both in HD patients and medical staff. However, the correction of increased ccf-gDNA levels was delayed in HD patients.

### Effect of isolation on the level of ccf-mtDNA

**Figure 3:**
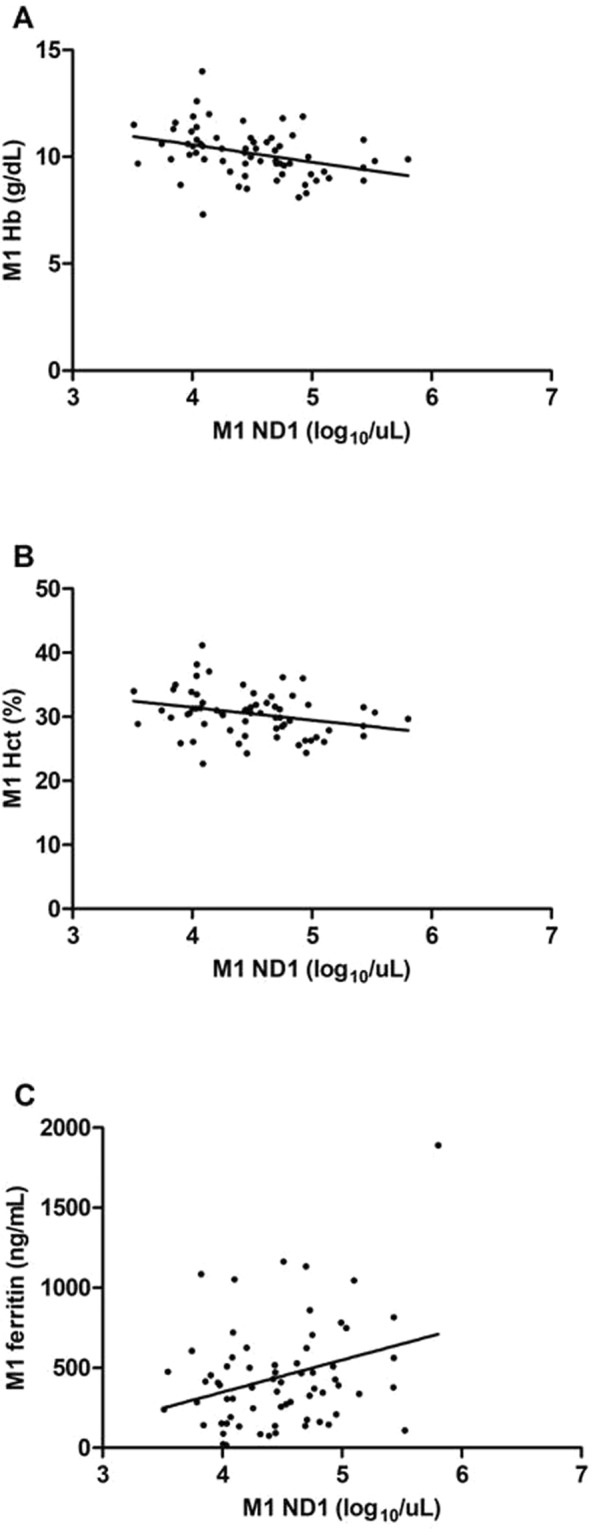
Correlation of clinical parameters and ccf-mtDNA at M1 (**A**,**B**) The level of ccf-mtDNA was negatively correlated with Hb (r = −0.345, *P* = 0.005), Hct (r = −0.279, *P* = 0.024) and (**C**) positively associated with ferritin (r = 0.302, *P* = 0.013). Pearson’s correlation test was used.

Similarly, ccf-mtDNA (ND1) was elevated at M0 and was not different between HD patients and medical staff (Fig. 2). At M1, the level of ccf-mtDNA was significantly lower in medical staff than in HD patients. However, ccf-mtDNA levels in HD patients only began to decrease after M1. The delta values of ccf-mtDNA between M0 and M1 were significantly higher in medical staff than in HD patients, but these values between M1 and M3 were lower in medical staff than in HD patients. Hb and Hct were negatively correlated, and ferritin was positively correlated with the levels of ccf-mtDNA at M1 in HD patients (Fig. 3). Levels of ccf-mtDNA were increased in both patients and medical staff, but processing was delayed in HD patients. An increase in ccf-mtDNA levels is associated with anemia and an acute reactive protein response.

### Effect of isolation on PTX-3

**Figure 4:**
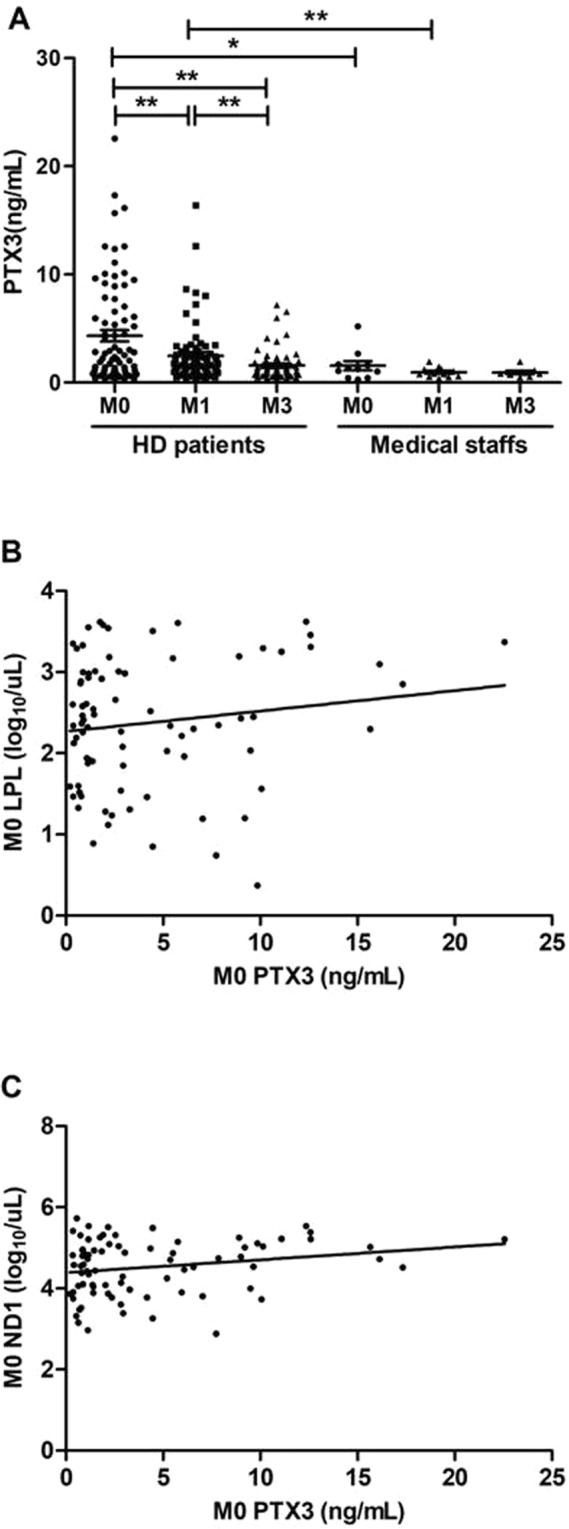
PTX-3 and correlation with the level of circulating cf-DNA (**A**) The level of PTX-3 was only increased in HD patients. It was highest at M0 and significantly decreased at M1 and M3. There was no difference in PTX-3 at M3 between the patients and medical staff. (**B,C**) PTX-3 was positively correlated with the level of ccf-gDNA (r = 0.484, *P* < 0.001) and ccf-mtDNA (r = 0.391, *P* < 0.001). *P value < 0.05 and ** < 0.01.

PTX-3 levels were markedly elevated at M0, and higher levels were maintained at M1 in patients than in medical staff. These levels were similar between HD patients and medical staff at M3 (Fig. 4). The levels of PTX-3 gradually decreased in HD patients from M0 to M3. When the patients were divided by average level of PTX-3 (4.4 ng/mL at M0), the high PTX-3 group showed higher levels of circulating cf-DNA at M0 and included more patients with DM-ESRD (Table 3). Ferritin was slightly increased in the high PTX-3 group, but this difference was not statistically significant. PTX-3 levels correlated positively with levels ccf-gDNA and ccf-mtDNA at M0. At M1 and M3, PTX-3 levels were positively associated only with ccf-gDNA (Supplementary Fig. S2). High PTX-3 group showed a more elevated level of circulating cf-DNA and included more DM-ESRD patients compared with low PTX-3 group.

**Table 3:** Characteristics of patients with high and low levels of PTX-3 at M0.

	High PTX-3 (≥4.4 ng/mL)	Low PTX-3 (<4.4 ng/mL)	*P* value
Age	64.56 ± 2.38	60.87 ± 1.97	0.238
Sex (M/F)	15/12	27/24	0.708
Dialysis vintage (months)	51.87 ± 11.02	41.39 ± 5.83	0.494
Charlson comorbidity index	4.59 ± 1.65	4.48 ± 1.97	0.806
**DM ESRD (%)**	**64%**	**36%**	**0.025**
SBP (mmHg)	145.10 ± 4.32	139.77 ± 2.26	0.282
DBP (mmHg)	74.24 ± 3.19	74.23 ± 1.55	0.999
ferritin (ng/mL)	572.22 ± 95.52	404.03 ± 41.54	0.063
**M0 PTX-3 (ng/mL)**	**9.75 ± 0.83**	**1.41 ± 0.14**	**<0.001**
**M0 ccf-gDNA (log**_**10**_ **/µL)**	**2.82 ± 0.15**	**2.19 ± 0.11**	**0.001**
**M0 ccf-mtDNA (log10/µL)**	**4.81 ± 0.13**	**4.41 ± 0.09**	**0.016**

DM ESRD, diabetes end stage renal disease; PTX-3, pentraxin-3; ccf-gDNA, circulating cell-free genomic DNA; ccf-mtDNA, circulating cell-free mitochondria DNA.

### Effect of isolation type on stress parameters

**Figure 5:**
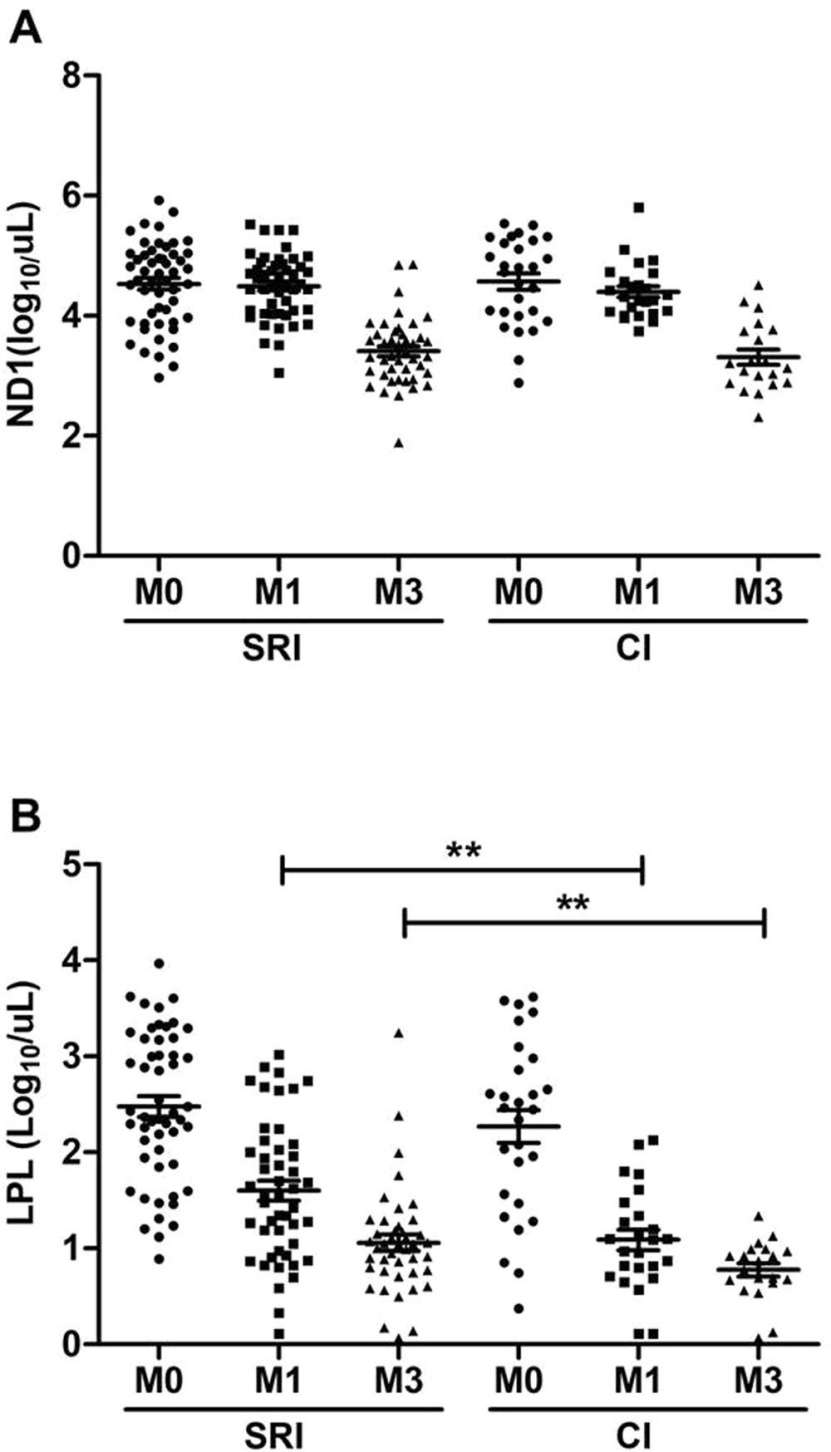
The change of circulating-cfDNA depending on isolation methods in HD patients The levels of circulating cf-DNA were similar in the patients on SRI and CI. However, (**A**) the level of ccf-gDNA was significantly higher in the patients on SRI at M1 and M3 than them on CI. (**B**) The level of ccf-mtDNA was not different depending on the method of isolation. SRI: Single room isolation, CI: cohort isolation *P value < 0.05 and ** < 0.01.

Fifty-four (65.1%) and 29 (34.9%) HD patients were hospitalized under single room isolation (SRI) and cohort isolation (CI), respectively. We aimed to determine whether the method of separation could affect stress parameters. Levels of ccf-gDNA were significantly increased in HD patients isolated under SRI at M1 and M3, although these levels were similar at M0 (Fig. 5). The levels of ccf-mtDNA were not different between the patients under SRI and CI.

## Discussion

A total of 83 HD patients and 12 medical staff in our hospital were isolated for 17 days because one patient was found to be infected with MERS. During a recent MERS outbreak in Saudi Arabia, many of infected patients were HD patients and medical staff in HD units and health care facilities^1^. Therefore, the quarantine of HD patients in our hospital was necessary for us to maintain dialysis and prevent additional infections. As an increasing number of people move easily between countries, dialysis units must be prepared in order to avoid the spread of infectious diseases while dialysis treatment continues.

We quarantined all of the HD patients in a single room to prevent the additional spread of MERS. Fifty-four HD patients receiving dialysis treatment at the same time or in the same space with the MERS patient was classified as the high-risk patients who could easily be contagious, and they had to be isolated even during dialysis. Additional dialysis machines were installed in hospital rooms for their dialysis. This quarantine for the high-risk patients was SRI. The remaining 29 patients who received dialysis treatment at a different time and in a different place with the confirmed MERS patient were also hospitalized. They continued dialysis treatment in an artificial kidney unit, with a 1-bed distance from the nearest patient. This quarantine was designated as CI. Fortunately, sputum examinations revealed that there were no newly diagnosed MERS patients in our dialysis unit. Our quarantine policy could have helped to prevent the further spread of MERS-CoV if there had been additional patients with MERS.

The staff required more time to prepare the dialysis treatment, including donning protection equipment and disinfecting the dialysis machines; thus, dialysis time was shortened slightly. Besides, the number of medical staff caring for the patients decreased, and face-to-face communication was limited. Laboratory findings showed decreases in Hb, Hct, and kt/v during quarantine although the change was subtle. It means the patients did not receive appropriate care. Considerable stress in itself could also have affected these clinical parameters. The levels of ccf-mtDNA as a stress marker were negatively correlated with those of Hb and Hct and positively associated with those of ferritin, an acute reactive protein. We had no sooner tried to correct anemia than MERS quarantine terminated. Significant increases in Hct at M3 reflected the effort. IS is protein-bound uremic retention that originates during intestinal fermentation^10^. IS concentrations were lower in HD patients maintaining a vegetarian diet compared with those in patients on a diet that included meat^11^. These results suggested that IS could be a nutritional marker. Our data also showed that the concentration of IS significantly increased after isolation. We speculated that the increase in IS levels related to nutrient recovery. HD patients and medical staff complained of poor meals and did not enjoy the food because they were provided only with lunch boxes. The increase in IS levels at M3 corresponded to the change of nutrition that accompanied the end of isolation.

This study found that circulating cf-DNA is abruptly elevated in not only patients but also medical staff. Living alone and working with protective equipment can cause high levels of mental and physical stress in not only patients but also healthcare workers during quarantine^12,13^. The result that the level of ccf-gDNA was higher in the HD patients under SRI reflected their severe stress. Levels of circulating cf-DNA steadily decreased in both patients and staff after isolation, but the decrease was delayed in the HD patients. Circulating cf-DNA in plasma indicates inflammation or cell death following cellular stress and tissue damage^3^. ccf-mtDNA containing CpG DNA repeats is known to attract neutrophils and activate immune cells^14–17^. Phagocytes quickly ingest the contents from apoptotic cells, therefore circulating cf-DNA is present in the circulation before phagocytes arrive^18^. When the engulfment of apoptotic bodies is reduced, or cell death is amplified, circulating cf-DNA can be substantially elevated^19^. Cellular apoptosis is common in HD patients due to chronic inflammation and immune dysfunction^20,21^. We suspected that more cells had undergone apoptotic and necrotic cell death or immune cells could not process circulating cf-DNA in HD patients. Also, chronic inflammation related to uremia, malnutrition, and calcification might have caused the late recovery of circulating cf-DNA. However, this study did not find a difference in the levels of circulating cf-DNA between HD patients and medical staff. Stress circumstances might be related to higher levels of ccf-mtDNA because chronic stress could alter the mitochondrial structure and lead to mitochondrial damage^22,23^. Healthcare workers, including nurses and medical doctors, are recognized as experiencing high levels of occupational stress^24^. Constantly elevated levels of circulating cf-DNA, especially ccf-mtDNA, are thought to reflect continuous occupational stress in our medical staff. We conducted a survey using Impact of Event Scale-Revised (IES-R) targeting the HD patients and medical staff at 12 months after the quarantine (Supplementary Fig. 3)^25^. The results identified the IES-R score at post-MERS was higher in medical staff than in HD patients. And post-MERS IES-R scores correlated positively with levels of ccf-mtDNA at M1. Healthcare workers responded with higher levels of psychological stress during the 2003 SARS outbreak^26^. They more easily recognize someone who will need to be hospitalized or die from infectious diseases. Thus, they might have worried more about becoming infected by MERS-CoV whenever they contacted patients. The higher IES-R scores in medical staff reflected the greater psychological impact and physical work stress. However, circulating cf-DNA was still not higher in HD patients than in medical staff at M3. If chronic inflammation was the only reason for the delay in processing circulating cf-DNA, it would be elevated in HD patients at M3. Therefore, insufficient care could be the main factor that caused the delayed recovery of circulating cf-DNA.

Our data showed that PTX-3 increased in HD patients only at the time of isolation, and decreased steadily. PTX-3 is an inflammatory cytokine sharing structural homology with CRP but is produced in response to proinflammatory stimuli^27^. It increases after HD and sensitively reflected in cardiovascular diseases and atherosclerosis^28,29^. More patients with DM ESRD were included in the high PTX-3 group. These results indicated that continuous inflammation tends to exist in diabetic patients regardless of acute stress. The patients with high PTX-3 levels showed significant elevation of circulating cf-DNA and mild increases in ferritin. There was a significant positive correlation between PTX-3 and circulating cf-DNA levels at M0, which was the acute phase of isolation. A previous study reported that the plasma from HD patients induced the release of interleukin-6 from peripheral blood monocytes of healthy controls^14^. We cautiously speculate that the stressful conditions caused by MERS increased circulating cf-DNA both in HD patients and medical staff but induced inflammatory responses in HD patients and not in medical staff.

In summary, stress parameters including circulating cf-DNA and PTX-3 showed that MERS quarantine had a critical effect on the HD patients and medical staff. However, elevated circulating cf-DNA levels slowly recovered in HD patients, and PTX-3 were only increased in HD patients. Laboratory findings revealed that HD patients had not been appropriately treated during quarantine and subnormal care might associate delayed recovery of circulating cf-DNA in HD patients. More attention to the improvement of HD patients is needed in such infection outbreaks. Moreover, plasma cell-free DNA and PTX-3 could be good indicators to evaluate stress and quality-of-care in HD patients.

## Methods

### Isolation strategy

Quarantine was started on June 16 and ended on July 2 in 2015. All HD patients were hospitalized in a single room for 17 days, but separated differently depending on their degree of infectious risk. Close contacts (n = 54) undergoing HD at the same time (3 sessions, from June 11 to 16) as the patient with MERS received isolated HD in their rooms installed with portable dialysis machines. We defined this quarantine as “single room isolation (SRI).” The rest of the patients (n = 29) were classified as low risk and received HD at artificial kidney units, with a 1-bed width distance from the neighboring patient. It was defined as “cohort isolation (CI).” Infection surveillance was performed using real-time PCR examinations of serial sputum samples obtained from HD patients and medical staff.

### Participants and plasma preparation

This study included a total of 95 participants, comprising 83 HD patients and 12 medical staff, at Gangdong Kyung Hee University Hospital. We collected 3 mL of whole blood in EDTA-coated tubes at 2, 4, and 16 weeks after isolation. Two weeks after the quarantine was included in the isolation period; therefore, we defined the sample taken at 2 weeks as “M0”. Samples obtained at 4 and 16 weeks after isolation were named M1 and M3, respectively. Briefly, whole blood was centrifuged at 1,500 × *g* at room temperature for 10 min, and the supernatants were transferred to fresh tubes. Plasma samples were stored at −80 °C and used after 12–16 months of storage. This study was approved by the Clinical Institutional Review Board of Gangdong Kyung Hee University Hospital, and written informed consent was obtained from all participants.

### Data collection

Demographic and clinical information was collected at the time of blood sampling at M0. The Charlson comorbidity index was calculated to assess the co-morbidity^30^. We obtained laboratory data, including serum hemoglobin (Hb), hematocrit (Hct), albumin, hsCRP, total cholesterol, single-pool kt/v, etc. for several months before and after isolation. Single-pool kt/v was calculated based on intradialytic blood urea reduction and weight change^31^. Sampling during the MERS quarantine resumed after the isolation was terminated; thus, the July sample was obtained four weeks after isolation. Accordingly, it was collected together with the sample to test for circulating cfDNA and PTX-3 at M1.

### Measurement of plasma indoxyl sulfate

Indoxyl sulfate was quantified by high-performance liquid chromatography (HPLC) with fluorescence detection^32^. The chromatographic equipment was an Agilent 1260 Infinity system (Agilent Technologies, Germany). The samples were prepared by mixing 250 µL of plasma with 900 µL of the internal standard solution, which was methyl paraben (100 µg/mL; Sigma-Aldrich) in acetonitrile (J.T. Baker; Deventer). Then, the samples were vortexed and centrifuged for 10 minutes at 3500 rpm, and 250 µL of supernatant was transferred to the HPLC system. The separation was performed on a Spherisorb OSD-2 C18 column (250 4.6 mm, 5 µm, Waters) with sodium acetate buffer (pH 4.5, Sigma-Aldrich, St. Louis, MO) and acetonitrile (10:90, v/v; J.T. Baker; Deventer) as the mobile phase at a flow rate of 1.3 mL/min. The excitation and emission wavelengths were set at 280 and 375 nm, respectively.

### Measurement of plasma cell-free genomic DNA and cell-free mitochondria DNA

Circulating cf-DNA was extracted from 200 μL of plasma using a QIAamp DNeasy Blood and Tissue kit (Qiagen, Valencia, Calif). The ccf-gDNA and ccf-mtDNA were amplified using a StepOnePlus real-time PCR system (Applied Biosystems, Massachusetts). Primers for the human NADH1 dehydrogenase 1 gene (ND1) were used for mtDNA, and primers for the human lipoprotein lipase gene (LPL) were used for gDNA. Standard DNA fragments of ND1 and LPL were synthesized using an Integrated DNA Technologies kit (IDT, Coralville, IA) for absolute quantification. The fragment solutions were 10-fold serially diluted. The concentrations of DNA were converted to copy number using the Andrew Staroscik Calculator for the absolute copy number from a template^33^. All samples were analyzed in duplicate, and a no-template negative control was included in every analysis.

### Pentraxin-3 (PTX-3) ELIZA

The level of PTX-3 in plasma was measured using a Quantikine ELISA Human Pentraxin 3/TSG-13 kit (R&D Systems, Minneapolis) according to the manufacturer’s protocol. The detection range was 0.31–20.00 ng/mL. Concentrations were determined using a standard curve generated from specific standards provided by the manufacturer. Each sample was measured in duplicate.

### Statistics

Statistical analyses were conducted using SPSS software (version 20 SPSS, Inc., Chicago, IL). For normally distributed variables, Student’s t-test and one-way ANOVA were used for comparisons, and the data are presented as the mean ± SE. The Mann-Whitney U and Kruskal-Wallis tests were used for comparisons of variables that were not normally distributed. Correlations were assessed with Pearson’s correlation coefficient for parametric distributions. Statistical significance was set at *P* < 0.05.

## Supplementary information

Dataset1
