# Supplementary material for: Inevitable isolation and the change of stress markers in hemodialysis patients during the 2015 MERS-CoV outbreak in Korea
Source: Sci Rep. 2019 Apr 5;9:5676. doi: 10.1038/s41598-019-41964-x (PMC6450937; doi:10.1038/s41598-019-41964-x)
Supplement: Supplementary file 1 — Dataset1 [file 41598_2019_41964_MOESM1_ESM.pdf]

Inevitable isolation and the change of stress markers in hemodialysis patients during the 2015 MERS-CoV outbreak in Korea

Yang Gyun Kim[1], Haena Moon[1], Se-Yun Kim[1], Yu-Ho Lee[1], Da-Wun Jeong[1], Kipyo Kim[1], Ju Young Moon[1], Young-Ki Lee[2], Ajin Cho[2], Hong-Seock Lee[3], Hayne Cho Park[4], Sang-Ho Lee[1*]

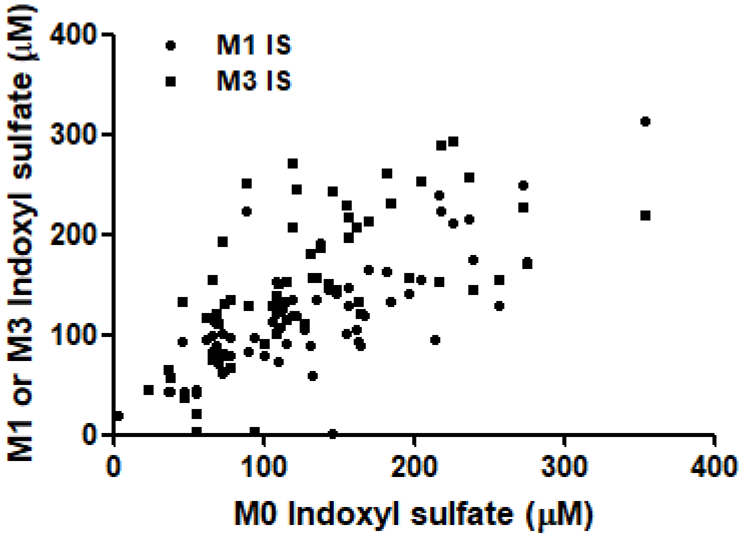

Supplementary Figure 1

Correlation of the level of M0 indoxyl sulfate with the levels of M1 or M3 indoxyl sulfate

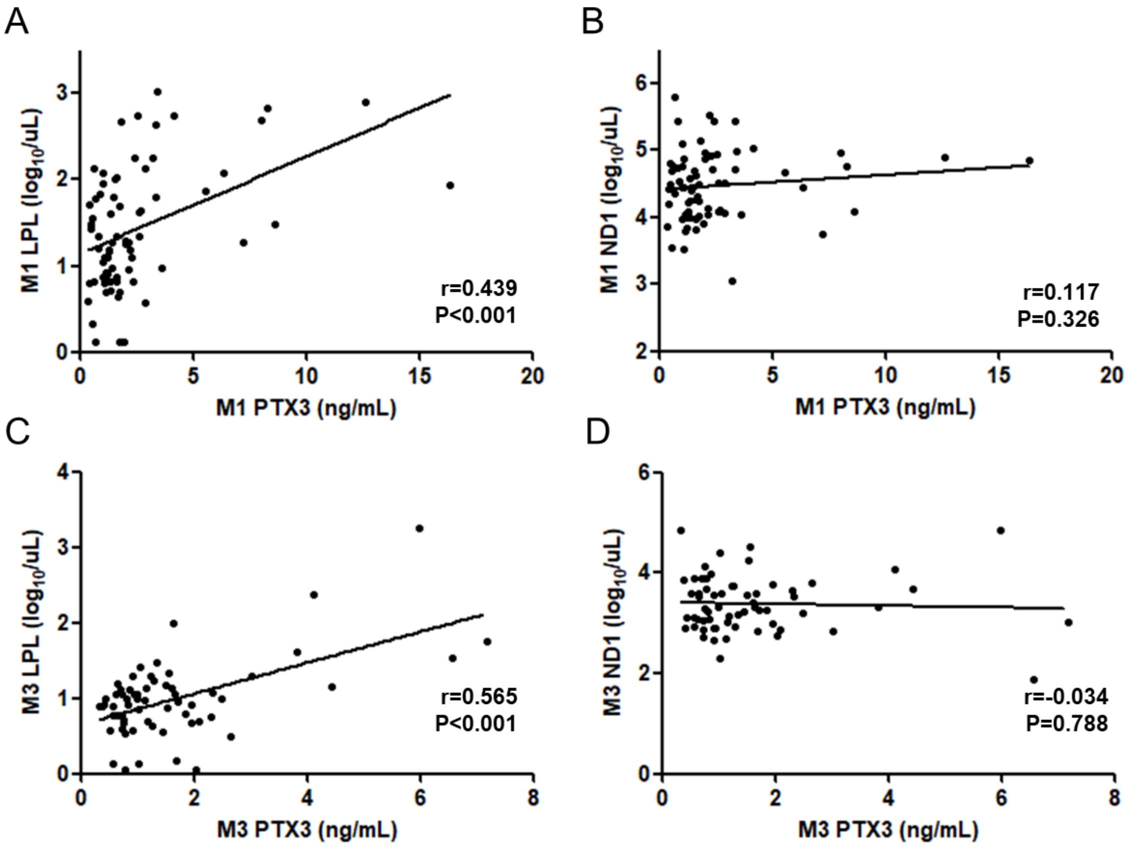

Supplementary Figure 2

The change of stress parameters depending on isolation methods in HD patients

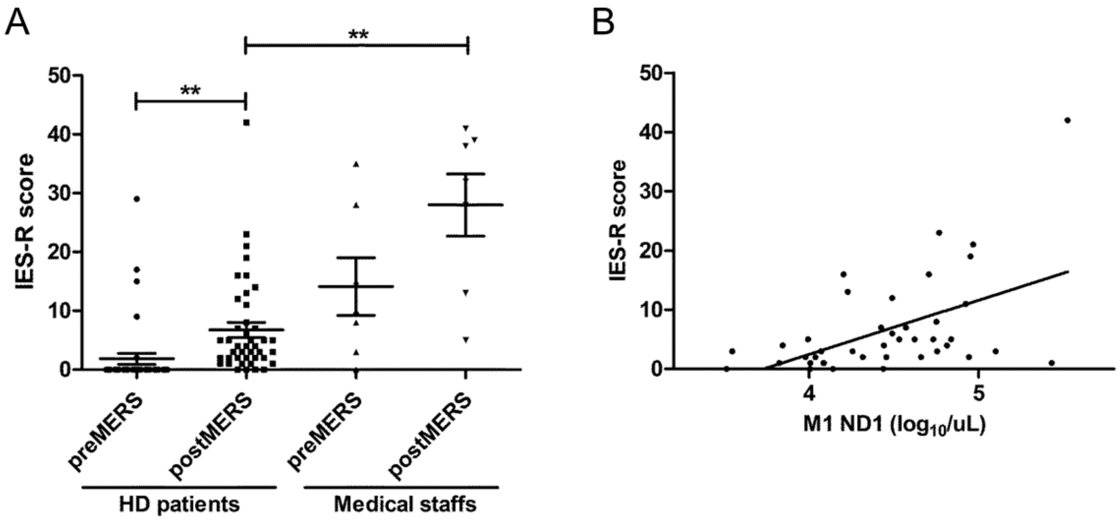

Supplementary Figure 3

Correlation of PTX-3 with ccf-gDNA and ccf-mtDNA at M1 and M3
